# Life-history traits and reduced variation of mass-reared black soldier fly (Diptera: Stratiomyidae) larvae fed dairy manure with *Rhodococcus* and *Paenibacillus* spp.

**DOI:** 10.1093/jee/toag088

**Published:** 2026-07-09

**Authors:** Amber E MacInnis, Branden Pierce, Dimitra Papantoniou, Heather R Jordan, Anjel M Helms, Jeffery K Tomberlin

**Affiliations:** Department of Entomology, Texas A&M University System, 2475 TAMU College Station, Texas, USA; Department of Biological Sciences, Mississippi State University, 295 Lee Blvd, 219 Harned Hall, Mississippi State, Mississippi, USA; Department of Entomology, Texas A&M University System, 2475 TAMU College Station, Texas, USA; Department of Biological Sciences, Mississippi State University, 295 Lee Blvd, 219 Harned Hall, Mississippi State, Mississippi, USA; Department of Entomology, Texas A&M University System, 2475 TAMU College Station, Texas, USA; Department of Entomology, Texas A&M University System, 2475 TAMU College Station, Texas, USA

**Keywords:** bioeconomy, *Hermetia illucens*, probiotic, waste digestion

## Abstract

Manure is an abundant waste source in dairy production with disposal creating significant challenges for producers. Recent work has shown that black soldier fly, *Hermetia illucens* (Linnaeus) (Diptera: Stratiomyidae), larvae can digest dairy manure; however, its high cellulose content and low nutrient value impact larval performance. Adding probiotics to black soldier fly feed can increase feed digestibility and weight gain. The goal of this study was to determine the influence of probiotic additions on black soldier fly larval performance when fed dairy manure. This study evaluated life-history traits of black soldier fly larvae fed dairy manure with single or combined probiotic treatments: *Rhodococcus* sp., *Paenibacillus* sp., and 1:1 combination, compared to a no-microbe control. Larvae were mass-reared on fresh dairy manure, and key metrics—bioconversion rate, survivorship, total biomass, and larval weight—were compared across treatments. Results showed a significant trial effect for all variables measured. Mean peak weights and the largest larval weights were 3.5-20.0% greater in at least one probiotic treatment compared to the control, while other life-history traits did not differ significantly. However, probiotic treatments reduced variation up to 17.0% in black soldier fly larval weight across trials, a trait not historically discussed in black soldier fly research. Developing processes that optimize larval weight in combination with reducing variation are critical for developing precise business models for predicting future production.

## Introduction

Animal production systems are a key component of agriculture with production expected to double by the year 2050 (Ilea 2009). Due to land-use constraints that limit the construction of new facilities, many producers are increasing the number and density of livestock in those currently existing (FAO 2006). This outcome is especially true for US dairy operations, which are concentrated in a few regions across the country. From the 1970’s to early 2000’s, the US herd size increased sixfold in existing dairies; and from 2000 to 2006, the number of farms with over 2,000 cattle doubled (MacDonald et al. 2007).

Dairy facilities produce large quantities of waste. On average, a lactating cow produces between 24.5 and 75.2 kg of manure daily (Nennich et al. 2005). Managing this waste presents a challenge for the industry. Some manure is used as fertilizer, but as dairy operations grow, this option only serves as a partial solution (Keplinger and Hauck 2006). Other practices include spreading solid waste for decomposition or use on neighboring farms as fertilizer (Van Horn et al. 1994). Other options include composting (Bernal et al. 2009) and anaerobic (Hills and Roberts 1981) digestion. Unfortunately, such methods can be cost-prohibitive.

Manure waste in excess can also result in negative environmental impacts. Land treated with excess manure can accumulate high levels of nitrogen and phosphorus that contaminate ground and surface water, resulting in eutrophication that harms aquatic organisms (Chang and Janzen 1996, McFarland and Hauck 1999, Glibert et al. 2005). This highlights the importance of developing more sustainable waste management approaches for dairy manure that reduce costs and limit environmental harm.

Black soldier fly, *Hermetia illucens* (Linnaeus), (Diptera: Stratiomyidae) larvae are generalists that can feed on a wide variety of decomposing organic materials, ranging from food waste to manure (Surendra et al. 2020, Broeckx et al. 2021). This ability to consume decaying organic waste is valuable for waste management. Furthermore, black soldier fly larvae fed an approved substrate can be used as an alternative protein source for a variety of animals (Diener et al. 2011), including swine (Newton et al. 1977), poultry (Pieterse et al. 2019), select fish species (Bondari and Sheppard 1987, Li et al. 2017, Fawole et al. 2020), and some pets (Kotob et al. 2022). Together, the ability of black soldier fly larvae to digest a variety of wastes and serve as animal feed provides a promising approach for improving sustainability in dairy and other livestock operations.

Recent research determined that black soldier fly larvae can efficiently digest and develop on different types of animal manure (Miranda et al. 2019). Black soldier fly larval digestion of manure has also been shown to reduce the amount of phosphorus, nitrogen, and dry matter (DM) in residual waste, sometimes greater than 50% (Myers et al. 2008). However, at an industrial scale, black soldier fly larval development was slower on dairy manure than on poultry or swine manure (Miranda et al. 2020). When black soldier fly larvae were fed contaminants (veterinary medicines, pesticides, heavy metals, mycotoxins, polyaromatic hydrocarbons, dioxins, and polycarbonated biphenyls) mixed with dairy manure, the levels for all contaminants in the larvae were below suggested safety concentrations set by the European Commission, World Health Organization, and Codex (Charlton et al. 2015). These data are promising and underscore the critical need for further studies on the safety of black soldier fly larvae fed on dairy manure, as well as new methods for improving black soldier fly larval performance for use as livestock feed.

Probiotics are live microorganisms that can confer health benefits when ingested by a host (Hill et al. 2014). Probiotics have been used in animal production to improve health and productivity for various livestock, including poultry (Patterson and Burkholder 2003), swine (Ross et al. 2010), cattle (Kelsey and Colpoys 2018), and fish (Zuenko et al. 2017). Probiotic treatments have been examined in some mass-reared insect species with the most common probiotics belonging to the genera *Lactobacillus, Streptococcus, Bacillus*, and *Saccharomyces* (Savio et al. 2022). For example, silkworm, *Bombyx mori* (Linnaeus) (Lepidoptera: Bombycidae) fed diets containing *Bifidobacterium bifidum* (Taha et al. 2017), *Lactobacillus acidophilus* (Suraporn et al. 2015), and *Saccharomyces cerevisiae* (Taha et al. 2017) had higher silk production. In silkworms, larval growth/weight was improved using *L. casei* (Suraporn and Terenius 2021) and two species of *Staphylococcus: Staphylococcus gallinarum* strain SWGB 7 and *Staphylococcus arlettae* strain SWGB 16 (Saranya et al. 2019).

Studies have also examined the influence of probiotic treatments on black soldier fly larval production. *Rhodococcus rhodochrous* 21198 is an oleaginous microbial species (Kooienga et al. 2020) with a high fat content that is capable of breaking down a number of recalcitrant compounds, including cellulose (a large component of dairy manure) with the potential to make substrates more nutritionally available to its host (Meng et al. 2009, Castro et al. 2016). The addition of *Rhodococcus rhodochrous* 21198 to a Gainesville diet (50% wheat bran, 30% alfalfa meal, 20% yellow corn flour), a control standard (Hogsette 1992) for US black soldier fly larvae production increased larval mass by 22% when reared at an industrial scale (Kooienga et al. 2020). At the benchtop scale, black soldier fly larvae fed a Gainesville diet supplemented with *R. rhodochrous* 21198 were twice as large and the conversion rate was twice that of the control diet (Franks et al. 2021).

Bacteria in the genus *Paenibacillus* were first classified in 1993 by Ash et al. using 16s rRNA sequencing to separate them from *Bacillus* (Ash et al. 1994, Alagawany et al. 2021). There are currently several hundred *Paenibacillus* species that have been described. *Paenibacillus* spp. are endospore forming bacilli that use peritrichous flagella for motility and have been isolated from a wide range of environments, including soils and bodies of water, as well as plant, insect, and human hosts (Shida et al. 1997). While several species are known pathogens to the organisms they inhabit, many have beneficial relationships with associated organisms with their positive effects on plant growth being particularly well studied (Grady et al. 2016). One such species, *Paenibacillus polymyxa*, has been demonstrated to be a promising option for biocontrol of pathogenic bacteria and fungi (Mülner et al. 2021). Most commonly associated with the rhizosphere or internal systems of plants, *P. polymyxa* has been isolated from sediments in bodies of water and can be found in fermented food products (Langendries and Goormachtig 2021).

Industrialization of *Paenibacillus* spp. has led to numerous applications. Several antimicrobial secondary metabolites that have been identified could be used to suppress multidrug-resistant pathogens or as a probiotic in livestock (Alagawany et al. 2021). Furthermore, some strains of this species produce enzymes for the degradation of lignin and cellulose, making it a promising means for the breakdown of waste streams high in plant matter into bioavailable nutrients for other organisms or for biofuel (Weselowski et al. 2016). Additionally, while some *Paenibacillus* spp. exhibit chitinase or crystal protein production (Ibrahim et al. 2014, Singh et al. 2016), *P. polymyxa* was found to increase aphid (Hemiptera: Aphididae) success (Kim et al. 2016), indicating that the presence of some strains of *P. polymyxa* may not directly negatively impact the growth of insects.

The purpose of this study was to evaluate larval life-history traits and bioconversion rate of black soldier fly larvae fed dairy manure amended with *Paenibacillus polymyxa*, a bacterial species also chosen due to its high cellulase activity, *R. rhodochrous* 21198, based on the previous work showing improvement with probiotic addition, and a 1:1 combination of both microbes.

## Materials and Methods

### Manure Preparation

Dairy manure was collected from Tarleton State University Dairy in Stephenville, TX, USA. The manure used in this study was less than 12 h old at the time of collection. Manure was collected into 18.9 L plastic buckets, and a lid was loosely placed over the top to allow some ventilation. The manure was transported to the Forensic Laboratory for Investigative Entomological Sciences (FLIES) Facility at Texas A&M University, College Station, TX, USA. The manure was homogenized in a 132.5 L plastic storage tote and strained with a fine mesh cloth until approximately 70% moisture content remained.

### Black Soldier Fly Larvae

The larvae used in this experiment were from a colony maintained at Texas A&M University FLIES Facility. Nursery units were prepared according to Tomberlin et al. (2023) with neonates that were less than 24 h old (Tomberlin et al. 2023). Two days prior to initiating the experiment, 10 nursery units were pooled together (approximately 130,000 larvae) and fed 300 g of Gainesville diet, which was hydrated to 70% moisture, and another 300 g the following day. The average larval weight of 500 (10 groups of 50) individuals was used to gravimetrically estimate the number of larvae for each treatment pan. Eggs and black soldier fly larvae were stored in a Rheem Environmental Chamber (Asheville, NC, USA) at 26 °C, 60% RH, and 16L:8D until the initiation of the experiment. Larvae used in all experiments were 7-9-d-old.

### Bacterial Cultures

Both the *Paenibacillus polymyxa* NCIB 8158 strain (ATCC 842) and the *Rhodococcus rhodochrous* 21198 strain (ATCC 372) were acquired from ATCC. *Rhodococcus rhodochrous* 21198 was grown on LB agar plates at 29 °C and in LB broth at the same temperature in shake incubators. *Paenibacillus polymyxa* NCIB 8158 was grown on LB agar and broth at 35 °C. Plates were scraped to harvest after 48 h, and the broth was spun down after 7 d of growth in an ultracentrifuge at 10,000×*g*, the supernatant was drained. Both agar and broth were used to harvest an appropriate amount of biomass to conduct the experiment with the plate primarily being utilized to better control for contamination. Collected bacteria were stored at 4 °C. Bacterial concentrations were chosen based on unpublished preliminary work.

For the first trial, bacterial cultures were enumerated through counting colony-forming units (CFU) from serial dilutions plated in triplicate. Concentrations of *R. rhodochrous* and *P. polymyxa* were determined to be 1.02 × 10^15^ CFU/ml and 5.80 × 10^14^ CFU/mL, respectively. These concentrated bacterial solutions were transported at 4 °C to the FLIES Facility at Texas A&M University, where they were diluted in phosphate-buffered saline (PBS). The *R. rhodochrous* 21198 solution was diluted to 5.08 × 10^13^ CFU/mL and the *P. polymyxa* solution was diluted to 5.8 × 10^13^ CFU/mL. Bacterial concentrations were further diluted by mixing 1.0 mL of each respective bacterial inoculant with 8.0 kg of strained dairy cattle manure, where final concentrations were 6.30 × 10^9^ CFU/g for *R. rhodochrous* 21198 and 7.25 × 10^9^ CFU/g concentration of *P. polymyxa*. Additionally, a solution of both bacterial species was created using a 1:1 mix of the two 10^13^ CFU/mL bacterial solutions, 1 mL of which was mixed into 8.0 kg of manure. The control manure received 1 mL of 1× PBS solution.

For the second trial, bacterial inoculant concentrations were 6.490 × 10^12^ CFU/mL for *R. rhodochrous* and 4.33 × 10^12^ CFU/mL for *P. polymyxa*. From these, 10.0 mL of the respective PBS- bacterial inoculant (7.0 mL bacterial inoculant in 3.0 mL PBS) was added to 8.0 kg of strained dairy cattle manure, yielding approximate bacterial concentrations of 5.67 × 10^9^ CFU/g *R. rhodochrous* and 5.41 × 10^9^ CFU/g *P. polymyxa*, respectively. A combination bacteria solution was created using a 3.5:5 ratio of *R. rhodochrous* to *P. polymyxa*, yielding a 5.41 × 10^12^ CFU/ml concentration. Ten milliliters of this mixed bacterial solution-PBS (8.0 mL bacterial mixture in 2 mL PBS) were added to 8.0 kg of strained manure, yielding an approximate concentration of 5.41 × 10^9^ CFU/g.

### Experiment Design

Black soldier fly larvae and diets were prepared following an industrial scale approach in 27 L clear Sterilite pans (Townsend, Massachusetts, United States) with approximately 10,000 larvae each. Each pan received 8 kg of strained dairy manure and the corresponding probiotic culture: *Rhodococcus*, *Paenibacillus*, or a 1:1 combination of *Rhodococcus* and *Paenibacillus* or PBS control with three replicates of each treatment per trial. Larvae were added immediately following inoculation. Based on preliminary data, additional time up to 3 d after probiotic inoculation did not impact life-history traits. A border of 0.8 kg dry Gainesville diet was added to each pan to prevent larval escape. Pans were placed in a randomized block design on metal shelves inside a Rheem Environmental Chamber (Asheville, NC, USA) at 29 °C, 60% RH, 16L:8D. Pan position was rotated daily to avoid position effects. At the start of the trials, three 5 g samples of manure from each replicate were collected and stored at −20 °C for moisture content. Mean larval weights were checked daily, starting 3 d after experiment initiation, by taking 30 of the largest larvae from a larval aggregation to minimize disturbance of the manure substrate. Larvae were weighed on a VWR-403B2 analytical scale (Radnor, PA, USA). Experiments were ended, and larvae were harvested once mean larval weights decreased or plateaued (within 5% of the previous day’s weight) for three consecutive days to ensure peak was obtained which varied across the treatments and replicates. Trials were conducted in September and October 2024.

At harvest, the total weight of each pan was recorded, and three 5 g samples of homogenized frass were collected from each replicate and stored at −20 °C for DM content analysis. Pans were then rinsed through a sieve three times to remove small solid material and capture larvae and remaining fibrous material. After rinsing, water was removed from the residual using a small mesh cloth and the residual was returned to a clean pan. Three, 5 g samples of larvae from each replicate were collected and stored at −20 °C for DM analysis. The remaining residual was weighed, and a 200 g subsample was sorted to estimate the total weight of larvae in the pan using the proportion of larvae in the subsample which is calculated by:


$$\frac{\mathrm{Weight}\;\mathrm{of}\;\mathrm{larvae}\;\mathrm{in}\;\mathrm{subsample}\;(g)}{\mathrm{Total}\;\mathrm{weight}\;\mathrm{of}\;\mathrm{the}\;\mathrm{subsample}\;(g)}$$

The total larval weight was estimated by:


Total pan weight (g) × proportion of larvae in subsample

All substrate samples (initial substrate, final frass, and final larvae) were thawed and placed in a weighed aluminum tin and dried in a Despatch LEB-1-75 drying oven (Lakeville, MN, USA) at 55 °C. The tins were weighed daily until the weight remained unchanged (within 5%). The largest larvae from the final harvest from each pan were dried using similar protocols. The three technical replicates for each pan were averaged. Percent DM was calculated using the following formula:


$$\frac{\mathrm{Weight}\;\mathrm{dried}\;\mathrm{sample}\;(g)-\mathrm{liner}\;(g)}{\mathrm{Initial}\;\mathrm{wet}\;\mathrm{weight}\;\mathrm{sample}\;(g)}\;\times \;100$$

Initial DM was calculated using the DM percentage and the weight of the starting diet (8 kg). After calculation, the weight of the dry border was added to the calculated DM value. The final DM was calculated using the final pan weight without larvae and the percent DM. Larval DM was calculated using the estimated larval weight and the percent DM. DM was calculated using the formula:


Total diet or larval weight (kg) ×DM(%)

The bioconversion rate (BCR) was determined using the DM from the initial diet set up and the larval DM at harvest for each replicate, which were then averaged for each treatment. Percent bioconversion was calculated by:


$$\frac{\mathrm{Initial}\;\mathrm{DM}\;(g)}{\mathrm{Larval}\;\mathrm{DM}\;(g)})\;\times \;100$$

Percent survivorship was calculated by using the estimated number of larvae at harvest divided by the estimated number of larvae at the start of the experiment and multiplying by 100%. Percent weight change was calculated by taking the average weight of the random sample (mg) at harvest, dividing by the average starting weight of larvae (mg) at the start, and multiplying by 100%.

Percent change in the coefficient of variation (CV) was calculated to quantify changes in the amount of variation compared to the control. This was calculated by using the standard deviation and the mean to calculate the CV for the probiotic treatments and the control. Then, the percent change in the CV from the treatment to the control was calculated by:


(CV probiotic treatment-CV manure only control) × 100%

The chosen variables, larval weight, bioconversion rate, survivorship, and total biomass produced, were chosen based on the applications to industrial production.

### Statistical Analysis

Data for percent dry matter, percent BCR, percent weight change, average weight of the largest at peak weight, average weight of the largest at harvest, average weight of the random sample at harvest, total biomass produced, and percent survivorship were collected for dried black soldier fly larvae produced from dairy manure treatments and control. Using a Shapiro-Wilk test, all variables were found to follow a normal distribution except percent weight change in the pooled trial, which was log transformed. For each variable, a two-factor analysis of variance (ANOVA) was utilized on pooled trial data using R studio (R v.4.2.1) (RStudio Team 2023) to determine the effects of trial and treatment. A Tukey HSD post hoc test was used on significantly different variables. Analyses for each trial were also run separately using the same methods as pooled data. Analysis of differences in weight over time between treatments was analyzed using a repeated measures ANOVA (RM ANOVA). Afterwards, a one-way ANOVA with a Bonferroni correction was utilized to evaluate the effect of treatment for each day. For all statistical tests, an alpha of 0.05 was used.

## Results

### Mean Weight Largest Larvae at Harvest

**Fig. 1.**
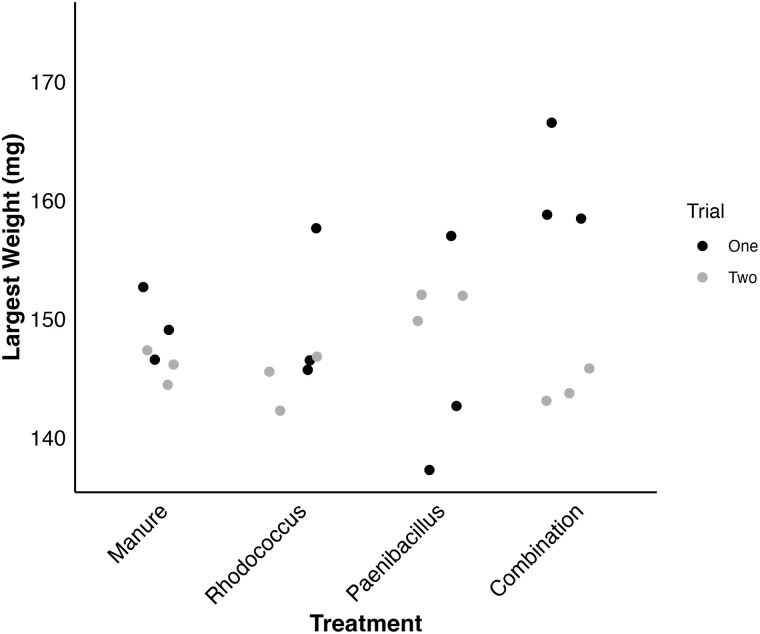
Dot plot for larval weight (mg) of the largest black soldier fly larvae at harvest for each treatment (manure control, manure with *Rhodococcus*, manure with *Paenibacillus*, and manure with a 1:1 combination of both microbes) for trial 1 (black) and trial 2 (grey) at 27 °C, 70% RH, and 14:10 L:D. There was a significant effect of trial. In trial 2, the *Paenibacillus* treatment was significantly heavier than the other treatments.

Using the pooled trial data, a significant (*F* = 5.49, df = 3, *P* = 0.01) trial by treatment interaction was determined for average weight for the largest individuals sampled. There was also a significant (*F* = 6.30, df = 1, *P* = 0.02) trial effect; therefore, trials were analyzed separately. In trial 1 (Fig. 1), the average weight of the largest larvae at harvest was not significantly (*F* = 3.06, df = 3, *P* = 0.09) different based on treatment. However, in trial 2 (Fig. 1), the average weight of the largest larvae at harvest in the *Paenibacillus* treatment was significantly heavier than the control (3.61%), *Rhodococcus* treatment (4.41%), and the combination treatment (4.90%).

### Percent Variation of Largest Larvae at Harvest

While the weight in trial 1 was not significantly different based on treatment, the percent variation in the average larval weight of the largest larvae at harvest increased for *Rhodococcus* (1.99%), *Paenibacillus* (4.04%), and the combination treatment (0.63%) compared to the control (Table 1). In trial 2 (Table 1), the percent variation in the average larval weight of the largest larvae at harvest increased in the *Rhodococcus* (0.53%) and combination treatments (0.02%) compared to the control, while the percent variation decreased in the *Paenibacillus* treatment (0.12%) compared to the control.

**Table 1.** Coefficient of variation and the percent change in the coefficient of variation for black soldier fly larval traits and BCR for each treatment (manure control, manure with *Rhodococcus*, manure with *Paenibacillus*, and manure with a 1:1 combination of both microbes) across trials at 27 °C, 70% RH, and 14:10 L:D. Positive numbers indicate increased variation, while negative numbers indicate decreased variation

	Weight largest		Weight random		Weight peak		BCR		Survivorship		Biomass		Weight change	
	CV	Δ CV (%)	CV	Δ CV (%)	CV	Δ CV (%)	CV	Δ CV (%)	CV	Δ CV (%)	CV	Δ CV (%)	CV	Δ CV (%)
**Trial 1**														
**Manure**	0.02	…	0.05	…	0.02	…	0.15	…	0.22	…	0.18	…	0.05	…
***Rhodococcus***	0.04	1.99	0.02	−2.9	0.03	0.1	0.1	−4.81	0.06	−16.33	0.05	−13.38	0.02	−3.05
***Paenibacillus***	0.06	4.04	0.03	−1.9	0.02	−0.64	0.08	−6.34	0.05	−17.41	0.02	−16.21	0.03	−1.99
**Combination**	0.02	0.63	0.04	−1.47	0.02	−0.26	0.22	7.52	0.11	−11.1	0.13	−4.45	0.04	−1.58
**Trial 2**														
**Manure**	0.01	…	0.04	…	0.02	…	0.16	…	0.10	…	0.13	…	0.04	…
***Rhodococcus***	0.01	0.53	0.02	−1.35	0.01	−0.50	0.09	−7.06	0.08	−2.21	0.07	−6.39	0.02	1.39
***Paenibacillus***	0.01	−0.12	0.03	−0.73	0.01	−0.82	0.10	−6.06	0.11	0.35	0.09	−3.76	0.03	−0.76
**Combination**	0.01	0.02	0.03	−0.83	0.02	0.03	0.03	−12.72	0.05	−5.29	0.04	−9.50	0.03	−0.85

### Mean Weight Random Larvae at Harvest

**Fig. 2.**
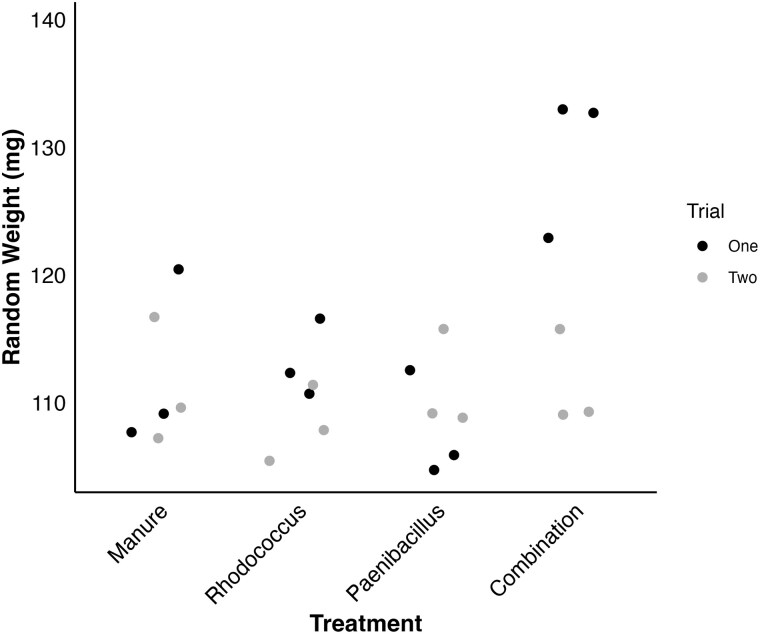
Dot plot for black soldier fly larval weight (mg) of a random sample of larvae at harvest for each treatment (manure control, manure with *Rhodococcus*, manure with *Paenibacillus*, and manure with a 1:1 combination of both microbes) for trial 1 (black) and trial 2 (grey) at 27 °C, 70% RH, and 14:10 L:D. There was a significant effect of trial. In trial 1, the combination treatment was significantly heavier than the other treatments.

Using the pooled trial data, a significant (*F* = 5.64, df = 3, *P* = 0.01) trial by treatment interaction was determined for average weight for a random sample of individuals at harvest. There was also a significant (*F* = 7.00, df = 1, *P* = 0.02) trial effect; therefore, trials were analyzed separately. In trial 1 (Fig. 2), the average weight of the random sample of larvae at harvest in the combination treatment was significantly heavier than the control (15.22%), *Rhodococcus* treatment (14.43%), and the *Paenibacillus* treatment (20.24%). However, in trial 2 (Fig. 2), the average weights for a random sample of larvae at harvest were not significantly (*F* = 0.436, df = 3, *P* = 0.734) different based on treatment.

### Percent Variation of Random Larvae at Harvest

In trial 1 (Table 1), the percent variation in the average larval weight of a random sample of larvae at harvest decreased for *Rhodococcus* (2.90%), *Paenibacillus* (1.90%), and the combination treatment (1.47%) compared to the control. In trial 2 (Table 1), the percent variation in the average larval weight of a random sample of larvae at harvest decreased for *Rhodococcus* (1.35%), *Paenibacillus* (0.73%), and the combination treatment (0.83%) compared to the control.

### Mean Weight Largest Larvae at Peak

**Fig. 3.**
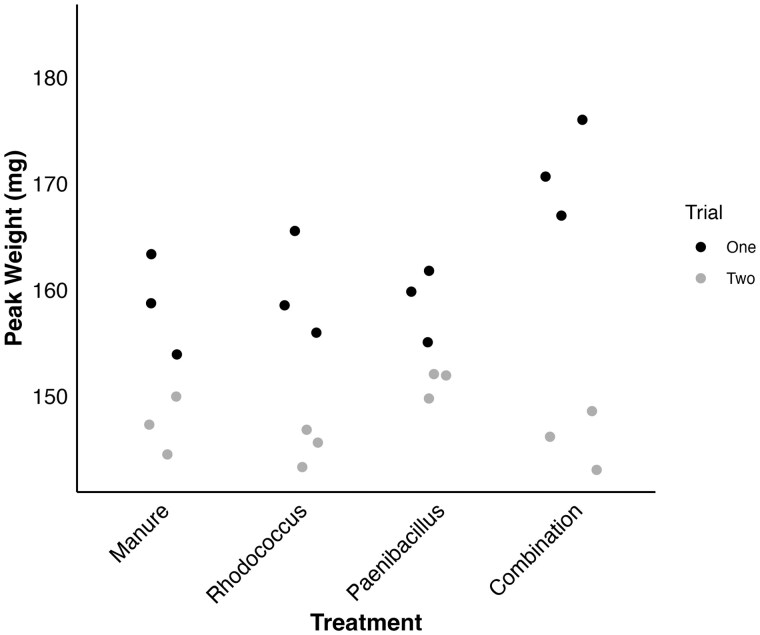
Dot plot for black soldier fly larval weight (mg) of the peak weight for each treatment (manure control, manure with *Rhodococcus*, manure with *Paenibacillus*, and manure with a 1:1 combination of both microbes) for trial 1 (black) and trial 2 (grey) at 27 °C, 70% RH, and 14:10 L:D. There was a significant effect of trial. In trial 1, the combination treatment was significantly heavier than the other treatments. In trial 2, the larvae in the *Paenibacillus* treatment were significantly heavier than the *Rhodococcus* treatment.

Using the pooled trial data, a significant (*F* = 7.02, df = 3, *P* < 0.01) trial by treatment interaction was determined for average weight for the largest individuals at peak weight. Therefore, trials were analyzed separately. In trial 1 (Fig. 3), the average weight of the largest larvae at peak in the combination treatment was significantly heavier than the control (7.89%), *Rhodococcus* treatment (7.01%), and the *Paenibacillus* treatment (7.76%). In trial 2 (Fig. 3), the average weight of the largest larvae at peak weight in the *Paenibacillus* treatment was significantly heavier than the *Rhodococcus* treatment (4.16%).

### Percent Variation of Largest Larvae at Peak

In trial 1 (Table 1), the percent variation in the average weight of the largest larvae at peak weight decreased for *Paenibacillus* (0.64%) and combination (0.26%), while the percent variation increased in the *Rhodococcus* treatment (0.10%) compared to the control. In trial 2 (Table 1), the percent variation in the average weight of the largest larvae at peak weight decreased for *Rhodococcus* (0.50%) and *Paenibacillus* (0.82%), while the percent variation increased in the combination treatment (0.03%) compared to the control.

### Mean Bioconversion Rate

**Fig. 4.**
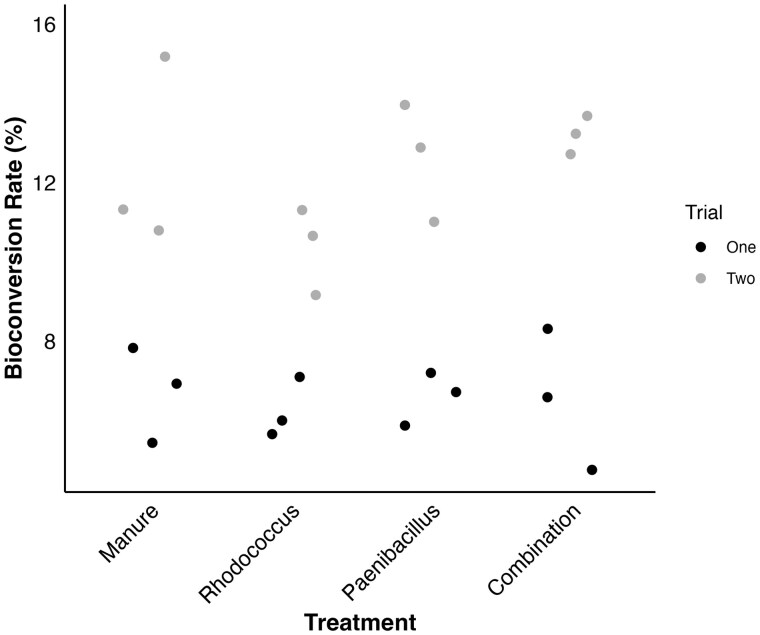
Dot plot for the bioconversion (BCR) rate (%) for each treatment (manure control, manure with *Rhodococcus*, manure with *Paenibacillus*, and manure with a 1:1 combination of both microbes) by black soldier fly larvae for trial 1 (black) and trial 2 (grey) at 27 °C, 70% RH, and 14:10 L:D. There was a significant effect of trial. There were no significant treatment effects in either trial.

Using the pooled trial data, a significant (*F* = 101.11, df = 1, *P* < 0.01) trial effect was determined for average BCR. Therefore, trials were analyzed separately. In trial 1 (Fig. 4), the average BCR was not significantly (*F* = 0.09, df = 3, *P* = 0.97) different based on treatment. In trial 2 (Fig. 4), the average BCR was not significantly (*F* = 1.94, df = 3, *P* = 0.20) different based on treatment.

### Percent Variation of BCR

In trial 1 (Table 1), the percent variation in the average BCR decreased for *Rhodococcus* (4.81%) and *Paenibacillus* (6.34%), while the percent variation increased in the combination treatment (7.52%) compared to the control. In trial 2 (Table 1), the percent variation in the average BCR decreased for *Rhodococcus* (7.06%), *Paenibacillus* (6.06%), and the combination treatment (12.72%) compared to the control.

### Mean Survivorship

**Fig. 5.**
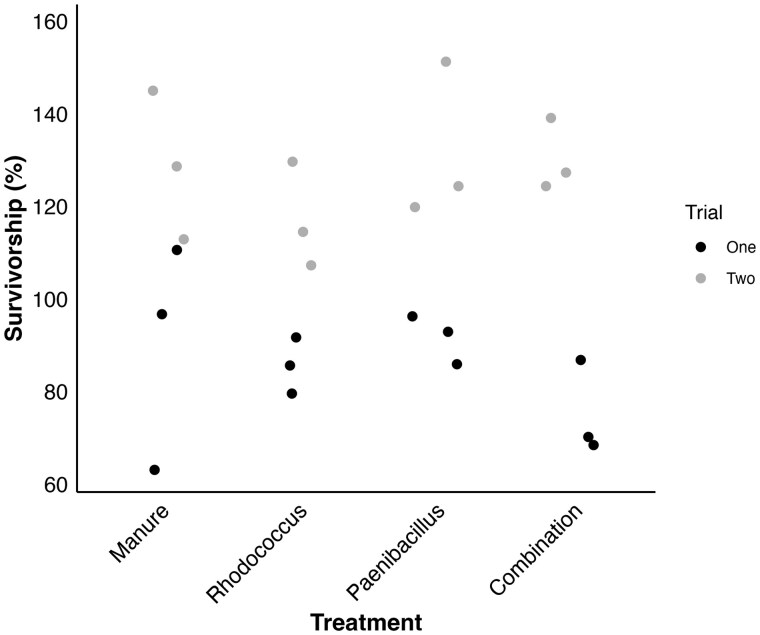
Dot plot for black soldier fly larval survivorship (%) for each treatment (manure control, manure with *Rhodococcus*, manure with *Paenibacillus*, and manure with a 1:1 combination of both microbes) for trial 1 (black) and trial 2 (grey) at 27 °C, 70% RH, and 14:10 L:D. There was a significant effect of trial. There were no significant treatment effects in either trial.

Using the pooled trial data, a significant (*F* = 54.71, df = 1, *P* < 0.01) trial effect was determined for average percent survivorship. Therefore, trials were analyzed separately. In trial 1 (Fig. 5), the average survivorship was not significantly (*F* = 0.87, df = 3, *P* = 0.50) different based on treatment. In trial 2 (Fig. 5), the average survivorship was not significantly (*F* = 0.73, df = 3, *P* = 0.56) different based on treatment.

### Percent Variation of Survivorship

In trial 1 (Table 1), the percent variation in the average percent survivorship decreased for *Rhodococcus* (16.33%), *Paenibacillus* (17.41%), and the combination treatment (11.10%) compared to the control. In trial 2 (Table 1), the percent variation in the average percent survivorship decreased for *Rhodococcus* (2.21%), the combination treatment (5.29%), while the percent variation increased in the *Paenibacillus* treatment (0.35%) compared to the control.

### Mean Black Soldier Fly Larval Biomass Produced

**Fig. 6.**
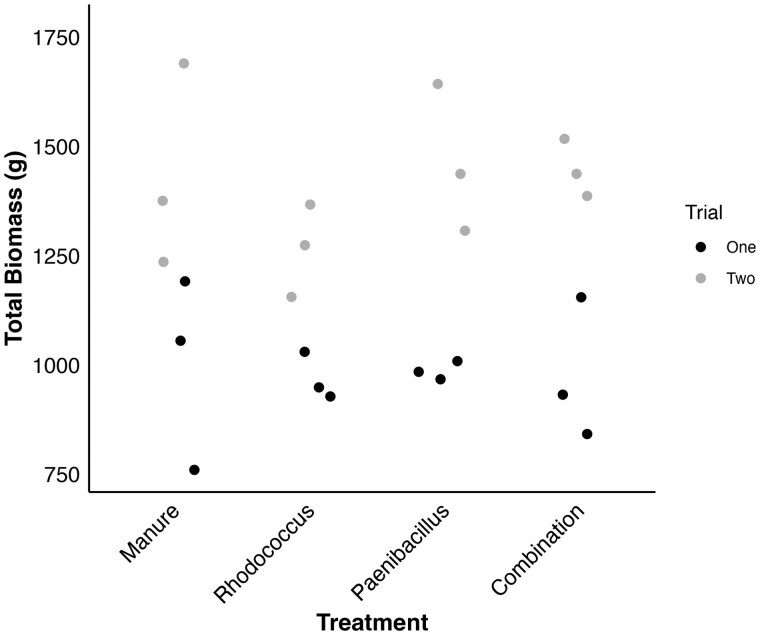
Dot plot for the total black soldier fly larval biomass produced (g) for each treatment (manure control, manure with *Rhodococcus*, manure with *Paenibacillus*, and manure with a 1:1 combination of both microbes) for trial 1 (black) and trial 2 (grey) at 27 °C, 70% RH, and 14:10 L:D. There was a significant effect of trial. There were no significant treatment effects in either trial.

Using the pooled trial data, a significant (*F* = 47.79, df = 1, *P* < 0.01) trial effect was determined for average black soldier fly larval biomass produced. Therefore, trials were analyzed separately. In trial 1 (Fig. 6), the average biomass produced was not significantly (*F* = 0.03, df = 3, *P* = 0.99) different based on treatment. In trial 2 (Fig. 6), the average biomass produced was not significantly (*F* = 1.03, df = 3, *P* = 0.43) different based on treatment.

### Percent Variation of Biomass Produced

In trial 1 (Table 1), the percent variation in the average biomass produced decreased for *Rhodococcus* (13.38%), *Paenibacillus* (16.21%), and the combination treatment (4.45%) compared to the control. In trial 2 (Table 1), the percent variation in the average biomass produced decreased for *Rhodococcus* (6.39%), *Paenibacillus* (3.76%), and the combination treatment (9.50%) compared to the control.

### Mean Percent Weight Change

**Fig. 7.**
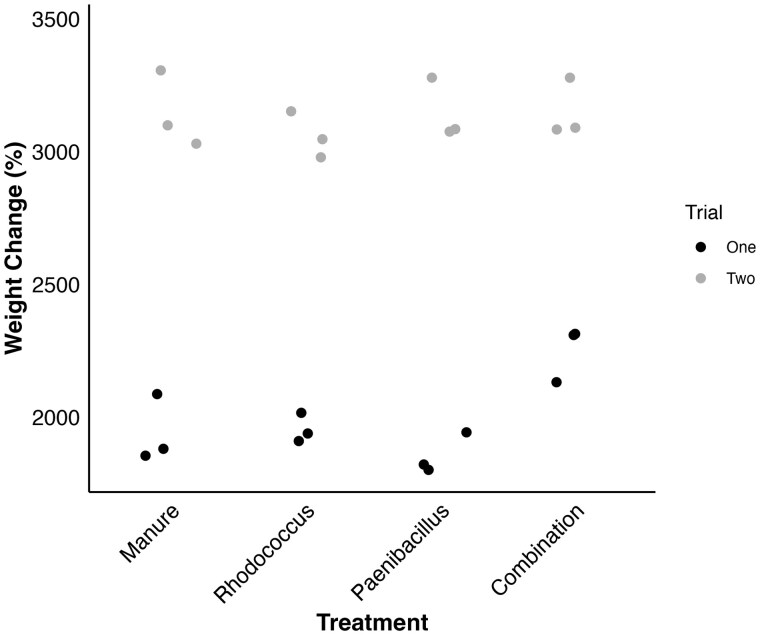
Dot plot for black soldier fly larval weight change (%) from start to finish of the experiment for each treatment (manure control, manure with *Rhodococcus*, manure with *Paenibacillus*, and manure with a 1:1 combination of both microbes) for trial 1 (black) and trial 2 (grey) at 27 °C, 70% RH, and 14:10 L:D. There was a significant effect of trial. In trial 1, the average percent weight change in the combination treatment was significantly higher than in the other treatments.

Using the pooled trial data, a significant (*F* = 5.71, df = 3, *P* = 0.01) trial by treatment interaction was determined for the average percent weight change of larvae from trial start to harvest. There was also a significant (*F* = 685.45, df = 1, *P* < 0.01) trial effect; therefore, trials were analyzed separately. In trial 1 (Fig. 7), the average percent weight change in the combination treatment was significantly greater than the control (16.01%), *Rhodococcus* treatment (15.17%), and the *Paenibacillus* treatment (21.33%). However, in trial 2 (Fig. 7), the average percent weight change was not significantly (*F* = 0.436, df = 3, *P* = 0.734) different based on treatment.

### Percent Variation of Weight Change

In trial 1 (Table 1), the percent variation in the average percent weight change decreased for *Rhodococcus* (3.05%), *Paenibacillus* (1.99%), and the combination treatment (1.58%) compared to the control. In trial 2 (Table 1), the percent weight change decreased for *Rhodococcus* (1.39%), *Paenibacillus* (0.76%), and the combination treatment (0.85%) compared to the control.

### Mean Weight Over Time

**Fig. 8.**
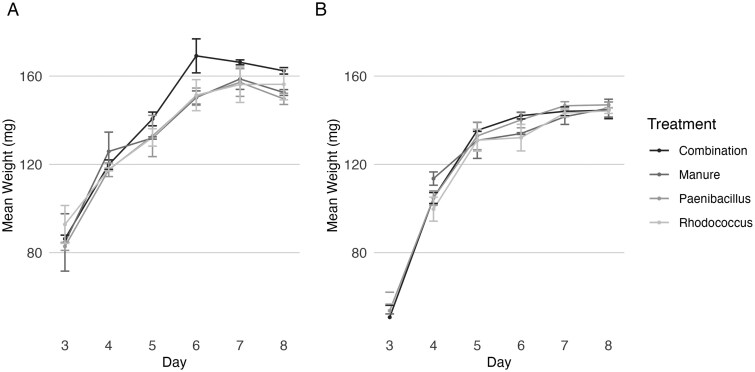
Line graph of the average weight of the largest black soldier fly larvae from each day (3 to 9) from trial 1 A) and trial 2 B) at 27 °C, 70% RH, and 14:10 L:D. Manure only control is in green, manure with *Rhodococcus* is light grey, manure with *Paenibacillus* is in grey, and manure with the 1:1 combination of microbes is black.

In trial 1 (Fig. 8A), RM ANOVA showed a significant (*F* = 1.84, degrees of freedom numerator (dfn) = 18, degrees of freedom denominator (dfd) = 48, *P* = 0.05) treatment by day interaction. The ANOVA to determine the effect of treatment for each day was not significant across any of the days. In trial 2 (Fig. 8B), RM ANOVA showed a significant (*F* = 2.44, dfn = 15, dfd = 40, *P* = 0.01) treatment by day interaction. The ANOVA to determine the effect of treatment for each day was not significant across any of the days.

## Discussion

Black soldier fly larvae are recyclers of many types of organic waste (Surendra et al. 2020, Broeckx et al. 2021), including a variety of manures (Miranda et al. 2019, El-Dakar et al. 2021). However, dairy manure is a suboptimal substrate for black soldier fly larvae due to limited nutrient availability and high cellulose content in manure (Li et al. 2016, Pelaez-Samaniego et al. 2017). Previous work adding *R. rhodochorous* 21198 to a Gainesville control substrate reported increased black soldier fly larval weight and a lower waste to larvae ratio (Kooienga et al. 2020, Franks et al. 2021). Additionally, both *Paenibacillus* and *Rhodococcus* are capable of digesting cellulose, which was hypothesized to increase the amount of substrate available for digestion for black soldier fly larvae (Bohra et al. 2019, Kooienga et al. 2020). In contrast, overall, for most measured variables, the findings in this study did not support a significant effect of *Paenibacillus* and *Rhodococcus* amendment to dairy manure on black soldier fly larval performance compared with the control (no microbes). However, an important discovery was the addition of these bacteria did decrease the variability in several black soldier fly larvae life-history traits compared to control (no microbe) substrates, suggesting a potential benefit for industrial applications to produce a more uniform product.

The finding that adding probiotics reduces variability in black soldier fly larval life-history traits, without a corresponding effect on performance, agrees with findings from other agricultural systems. For example, piglets fed *Lactobacillus reuteri* BSA131 showed reduced growth variability compared with those fed control diet, despite no difference in total weight gain across treatments (Chang et al. 2001). In another study, weaned piglets fed a combination of three probiotics, *L. plantarum* ATCC 4336, *L. fermentum* DSM 20016, and *Enterococcus faecium* ATCC 19434, had higher growth and lower variability in growth at the highest dose tested (Veizaj-Delia et al. 2010). These results indicate a benefit of probiotic treatments in the industry to create more uniform and faster-growing swine. In this system, swine were given an optimal diet, whereas our black soldier flies were given a suboptimal diet, which could be why we did not see significant differences in the measured traits. Reducing variability in any system creates a more uniform, predictable product.

The initial inoculum of bacterial amendment used can be an important factor influencing animal performance and can be microbial and animal species and strain dependent. In this study, we tested a single concentration of probiotics, and future work should examine the effect of dose on the life-history traits of black soldier fly larvae fed dairy manure. In broiler chickens, outcomes were strain and concentration-specific depending on the probiotics used. While both low and high concentrations were tested, only low concentrations of the bacteria *L. casei* and high concentrations of *L. acidophilus* significantly improved broiler weight and weight gain. For the fungus *Scytalidium acidophilum*, only high fungal concentrations in feed significantly improved broiler weight, broiler weight gain, and feed intake (Huang et al. 2004). Given the studies with both broiler chickens and swine, it is possible that both our strain of *Rhodococcus* and *Paenibacillus* could have similar dose response effects which were not examined in this study but should be examined in the future. Preliminary unpublished work with *Rhodococcus* examining dose and pretreatment time of substrates found that with higher concentrations and no pretreatment time, there was no effect on measured traits, in combination with a longer pretreatment time, bioconversion increased in *Rhodococcus* only. The impact on survivorship could not be addressed due to the estimated larval numbers and the moisture content of the substrates dried out during pretreatment time, leading to negative effects on all other combinations. Due to the negative impacts seen with other microbial treatments, the lower inoculum concentration without a pretreatment time was chosen. Future work should continue to explore the impact of pretreatment time and inoculum concentration with probiotic applications in terms of regulating substrate moisture for larval performance.

There was a significant trial effect across all measured variables despite the studies being conducted only one month apart (in September and October 2024), with manure from the same dairy. Samples were collected during the early morning hours and in Stephenville, TX for trial 1 the low temperature was 22.4 °C while for trial 2 the low temperature was 20.7 °C. Temperature differences or other abiotic factors could cause changes in the microbial communities of the starting material. This is currently being analyzed in a separate study and could have contributed to the increased variability seen across trials. Finally, the starting moisture content of the manure in trial 1 was slightly lower at an average of 65% compared with trial 2, which had an average of about 75% moisture. The average final weight or both the random and largest larvae were larger in the first trial than in the second also with lower survivorship in trial 1. With the lower survivorship of larvae, the remaining survivors now have fewer larvae competing for the same amount of resources and thus can grow larger. This has been demonstrated with black soldier fly in density studies where lower densities of larvae develop faster, and individuals are larger (Diener et al. 2009, Jones and Tomberlin 2019). The lower survivorship in trial 1 could be linked to lower initial moisture content. Moisture content is an important factor in black soldier fly larval development where larvae have been shown to develop up to 5 d faster with 10% higher moisture content and were up to 40 mg heavier (Cheng et al. 2017). Future work should examine the influence of abiotic factors, including the influence of starting moisture, on the performance of black soldier fly larvae in challenging substrates such as dairy manure.

This study was conducted at an industrial scale, which required some estimation of some of the biomass of larvae produced. Error in the estimation of the total amount of larval biomass produced could be responsible for the lack of treatment effects seen in this study. Additionally, the high level of variation between trial could be due to low replication within each trial (3 replicates per trial) and the estimation of some of the variables (ie survivorship and bioconversion rate). A smaller-scale bench-top study with hand-counted larvae at the start and end would allow for further refinement of these measured variables and increased replication. The high variability between treatments may also be caused by the poor nutritional quality of the diet. Dairy manure is a suboptimal rearing substrate mainly due to the high crude fiber content (Li et al. 2016). In substrates that are nutritionally poor, CV increased for wing shape, size, and sternobristle number *Drosophila melanogaster* Meigen (Diptera: Drosophilidae) (Takahashi et al. 2011). Nutritional stress is thought to increase the variation for morphological traits (Imasheva et al. 1999). Another explanation for the increased variation could be due to the plasticity of the organisms during development. In the cases of poor food quality, larvae may extend their feeding time (Krittika et al. 2019) to reach the critical weight for pupation increasing the observed variability. This response would prioritize surviving rather than reaching optimal size. Also, poor quality diets are capable of shifting the microbiome of organisms, which could result in changes in nutrient absorption, leading to variability in size (Xie et al. 2015).

However, determining the impact of such treatments is of value for optimizing production, while reducing variation—a trait historically not discussed in previously published research. Indeed, the reduction in variation in black soldier fly larval production is important as it creates a more predictable product. Based on data recorded, using probiotic treatments in black soldier fly larval digestion of dairy manure can be used to significantly increase larval weight in conjunction with reduced variation. Greater precision and accuracy with such processes could lead to developing business models that reliably estimate output (ie insect biomass), which would lead to more accurate price points for the market.
